# Transient Expression Vector Construction, Subcellular Localisation, and Evaluation of Antiviral Potential of Flagellin BP8-2

**DOI:** 10.3390/molecules29163876

**Published:** 2024-08-16

**Authors:** Yahan Chen, Jianxin Zhong, Meihuan Lu, Chengde Yang

**Affiliations:** 1 College of Plant Protection, Gansu Agricultural University, Lanzhou 730070, China; 17693338867@163.com (J.Z.); yangcd@gsau.edn.cn (C.Y.); 2 Microbial Resources of Research Center, Microbiology Institute of Shaanxi, Xi’an 710043, China; lu_meihuan@sina.com

**Keywords:** flagellin BP8-2, transient expression, subcellular localisation, TMV, inhibitory

## Abstract

This study used the DNA of *Bacillus amyloliquefaciens* Ba168 as a template to amplify the flagellin BP8-2 gene and ligate it into the fusion expression vector pCAMBIA1300-35S-EGFP after digestion for the construction of the expression vector pCAMBIA1300-EGFP-BP8-2. Next, using *Nicotiana benthamiana* as receptor material, transient expression was carried out under the mediation of *Agrobacterium tumefaciens* C58C1. Finally, the transient expression and subcellular localisation of flagellin BP8-2 protein were analysed using the imaging of co-transformed GFP under laser confocal microscopy. The results showed that flagellin BP8-2 was localised in the cell membrane and nucleus, and the RT-PCR results showed that the BP8-2 gene could be stably expressed in tobacco leaf cells. Furthermore, there was stronger antiviral activity against tobacco mosaic virus (TMV) infection in *Nicotiana glutinosa* than in BP8-2 and ningnanmycin, with an inhibitory effect of 75.91%, protective effect of 77.45%, and curative effect of 68.15%. TMV movement and coat protein expression were suppressed, and there was a high expression of *PR-1a*, *PAL*, and *NPR1* in BP8-2-treated tobacco leaf. These results suggest that flagellin BP8-2 inhibits TMV by inducing resistance. Moreover, BP8-2 has low toxicity and is easily biodegradable and eco-friendly. These results further enrich our understanding of the antiviral mechanisms of proteins and provide alternatives for controlling viral diseases in agriculture.

## 1. Introduction

Bacteria are widely distributed organisms and characterised by rapid propagation and diverse biological activities, and microorganisms play an important role in nature [1,2,3]. These bacteria produce metabolic products (e.g., secondary metabolites) that may be promising for agricultural applications [4]. Many types of bioactive components have been screened and isolated from some bacteria, which can inhibit plant viruses [5,6,7,8]. Flagellin, the most highly expressed structural protein, is the primary constituent of flagellated bacteria [9,10]. It was initially hypothesised to be involved in bacterial motor invasion; however, discoveries indicate that flagellins have different structures, sizes, and functions in different bacterial strains and that different flagellins have different immune functions within the same bacterium [11]. Flagellin has excellent adjuvant activity that induces effective immune responses in humans. As an activator protein, it induces plant resistance to various pathogens and insects [12,13]. Flagellin BP8-2 was identified in *Bacillus amylolyticus* and exhibits antifungal activity against *Penicillium expansum* and *Verticillium dahliae* [14,15]. BP8-2 is safe, easily biodegradable, and eco-friendly [16,17].

Transient expression pertains to the technique of directly introducing foreign genes into cells in a non-integrated manner to attain a high level of gene expression within a brief period [18]. Owing to transient expression not requiring the re-integration of foreign genes into the chromosomes of cells, it neither entails repeated screening nor yields stable genetic offspring. In contrast with stable expression, transient expression has traits such as simplicity, rapidity, a short cycle, high efficiency, biosafety, and stability [19]. Hence, this technology has been extensively employed in the domain of molecular biology research and the research and development of vaccines, antibodies, and biopharmaceuticals [20,21]. The Agrobacterium-mediated approach is the most prevalent and extensively used method for introducing foreign genes into plants; its advantages are straightforward operation, facile transfection, and high expression efficiency [22]. This approach can carry a considerable number of foreign gene fragments and achieve the transfer and integration of foreign genes into host plant cells with the assistance of Agrobacterium infection [23]. Subcellular localisation refers to ascertaining the specific position where the final product of gene expression is located in tissue cells, that is, predicting the subcellular location based on the given protein sequence structure [24]. Currently, the optimal method for verifying subcellular localisation is laser technology grounded in traditional immunofluorescence technology and fusion protein technology [25]. Predicting the subcellular localisation of BP8-2 is critical for comprehending the fundamental properties and functions of protein structure and the interaction between protein and plant cells, and investigating the mechanism of disease and its rules and mysteries.

Plant virus diseases are one of the most destructive crop diseases, often known as ‘plant-cancer’ [26,27]. Annually, an estimated economic loss worth several billion dollars is encountered worldwide due to the prevalence of plant viruses in agricultural fields [28]. In recent years, economic losses have been increasing due to the impact of climate anomalies and crops affected by viral diseases, especially in China [29]. Tobacco mosaic virus (TMV), the type species of the genus *Tobamovirus* and a member supergroup alphavirus-like, was first described by Mayer in 1886 [30]. It has a positive single-stranded RNA genome that is encapsidated into a rod-shaped particle of about 300 nm in length and 18 nm in diameter. TMV has a broad host range of weed and crop species, including tobacco, pepper, and tomato that can be infected experimentally, and is distributed worldwide [31]. Currently, curing plants infected by viruses is not feasible, and no pesticides effectively treat them. Therefore, the development of efficient, environmentally friendly antiviral agents is urgently required.

In this study, using the DNA of *Bacillus amyloliquefaciens* Ba168 as the template, the flagellin BP8-2 gene was amplified using PCR and then ligated into the fusion expression vector pCAMBIA1300-35S-EGFP after digestion to construct the expression vector pCAMBIA1300-EGFP-BP8-2. Next, using *Nicotiana benthamiana* as receptor material, transient expression was carried out under the mediation of *Agrobacterium tumefaciens* C58C1. Finally, the transient expression and subcellular localisation of flagellin BP8-2 protein were analysed using imaging of co-transformed GFP under laser confocal microscopy. This study also investigated the anti-virus effect of BP8-2 and its possible mechanisms in controlling tobacco mosaic virus (TMV) in tobacco. These results further enrich the understanding of the antiviral mechanisms of proteins and provide alternatives for controlling viral diseases in agriculture.

## 2. Results

### 2.1. Amplification and Identification of BP8-2 Gene

Using the extracted Ba168 genomic DNA as a template, PCR amplification was performed with specific primers with enzyme cut sites, and specific bands of ~840 bp in length were obtained, consistent with the expected size. The target sequence strips were recovered using a gel and connected with the pMD19-T carrier. DH5α receptor cells were transformed using the link product, and the positive clones were identified and sequenced using Amp-resistant plate screening and bacterial solution PCR. The sequence comparison analysis was consistent with the results, indicating that the cloning of the BP8-2 gene was successful.

### 2.2. Construction and Sequencing of Recombinant Plasmid

Plasmids extracted from the empty carrier and positive culture were digested with the restriction endonuclidene enzymes KpnⅠ and XbaⅠ, and the digested products were identified and sequenced using 1% agarose gel electrophoresis. The size of the fragment cut by the recombinant plasmid was the same as the length of the BP8-2 gene sequence, and subsequent sequencing analysis was consistent with the results. The DH5a cloned strain plasmid was linked to the BP8-2 gene.

### 2.3. Subcellular Localisation and Transient Expression of BP8-2

**Figure 1:**
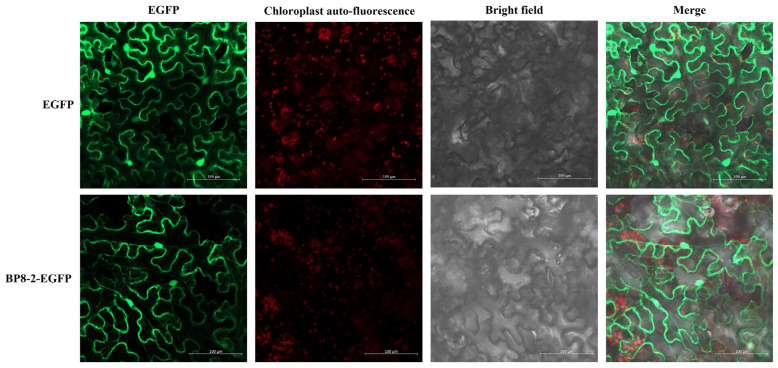
Subcellular localisation of flagellin BP8-2 in Benthamite.

**Figure 2:**
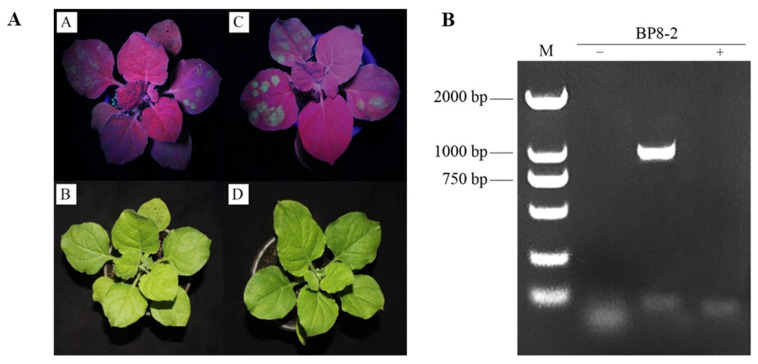
RT-PCR validation of transient expression of gene BP8-2. (**A**) BP8-2 gene transient expression (A, B is empty; C, D is BP8-2 treatment); (**B**) BP8-2 gene PCR validation (M: 2000 DNA Marker; −: negative control; +: empty).

The empty 35S::EGFP vector and the pCAMBIA1300-EGFP-BP8-2 recombinant vector were transformed into the leaves of tobacco via the Agrobacteria-mediated transient expression system and then expressed in the leaf epidermal cells of tobacco. After 3 d, the upper epidermis of the leaves was removed and observed using confocal laser microscopy. The green fluorescence of the empty 35S::EGFP vector was detected in the cytoplasm and the nucleus of the epidermal cells of the upper epidermis of the tobacco, indicating that the subcellular localisation vector could be expressed normally. Moreover, the green fluorescence of BP8-2 in the nucleus and cell membrane was distinctly visible. The target protein was localised in the cell membrane and nucleus (Figure 1). Total RNA extracted from the upper leaves of the treatment group and the control group was collected 9 d after injection and verified using RT-PCR, suggesting that the target gene can be stably expressed in tobacco plants and indicating accurate subcellular localisation results (Figure 2).

### 2.4. Purification of BP8-2 Proteins

**Figure 3:**
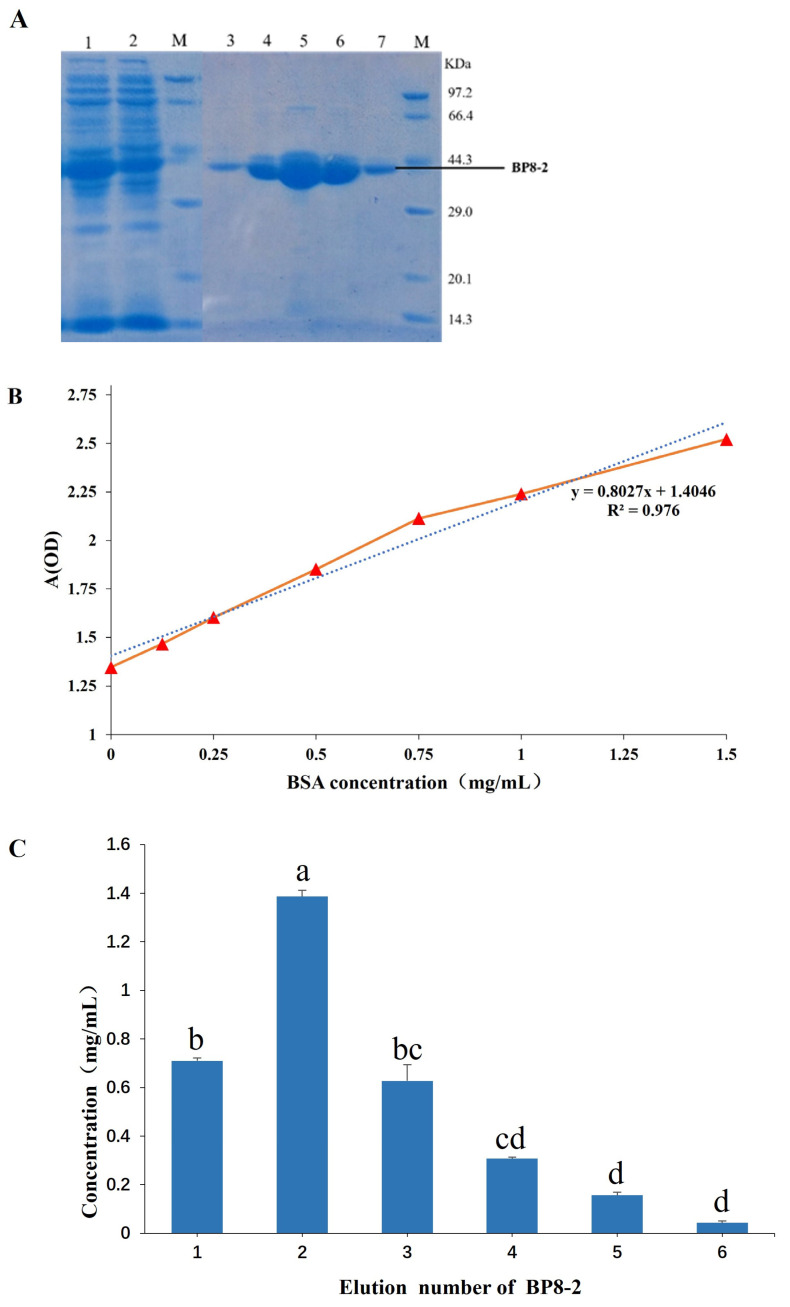
Sodium dodecyl sulphate-polyacrylamide gel electrophoresis SDS-PAGE detection (**A**) and purified protein concentration determination (**B**,**C**) of BP8-2. (**A**) M: protein marker; lane 1–2: induced recombinant bacteria lysis supernatant; lane 3–7: elution of 1–5-fold purified protein eluate.; (**B**) protein standard curve. Blue dashed line is regression straight line; (**C**) change in eluate concentration for different elution times. Different lowercase letters indicate significant difference at *p* < 0.01 level by Duncan’s new multiple range test.

As shown in Figure 3A, SDS-PAGE electrophoretic analysis showed that a prominent protein band of ~40 kDa was evident on the gel, and there was no distinct difference between total cell and supernatant fractions, indicating that the protein was highly stable and remained soluble in the sonication buffer. After Ni+ affinity chromatography, the target protein band was single, and the purification effect was good. The Bradford method was used to determine protein concentration, the absorbance value was measured using the protein standard solution (20 mg/mL BSA) to dilute different concentration gradients under the ELISA instrument, and the standard curve was drawn (Figure 3B). The absorbance value of the target protein (A595) was substituted into the linear regression equation to obtain BP8-2 concentration. The results showed that the maximum amount of target protein was 1.32 mg/mL after two elution times, consistent with the results of SDS-PAGE electrophoresis (Figure 3C).

### 2.5. Anti-TMV Activities of BP8-2

**Figure 4:**
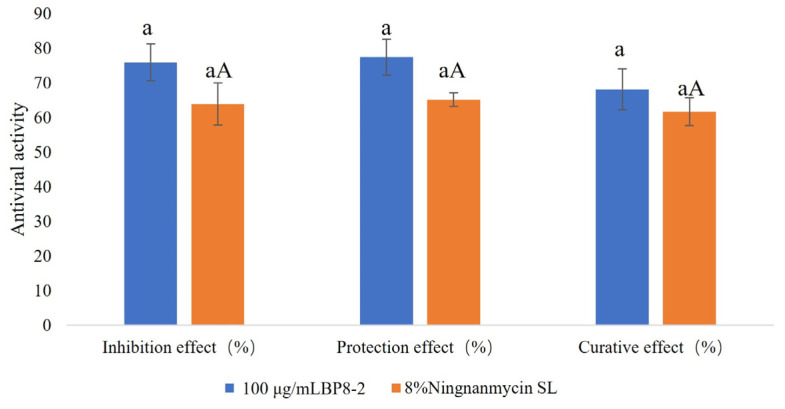
Antiviral activity of protein BP8-2 against TMV. Different uppercase or lowercase letters indicate significant difference at *p* < 0.01 level by Duncan’s new multiple range test.

The inhibition effect, protection activity, and curative effect of BP8-2 were tested at the concentration of 100 μg/mL in *Nicotiana glutinosa* by using the half-leaf method. The results of curative, protective, and inactivation efficacies are presented in Table 1 and Figure 4. Based on the inhibition rates of local lesions on the leaves of *N. glutinosa*, the antiviral activity of BP8-2 was more than that of 1000 times 8% ningnanmycin SL, with an inhibitory effect of 75.91%, protective effect of 77.45%, and curative effect of 68.15%. These results suggest that BP8-2 has a significant inhibitory effect on TMV infections.

### 2.6. Effects of BP8-2 on TMV Infection Movement

**Figure 5:**
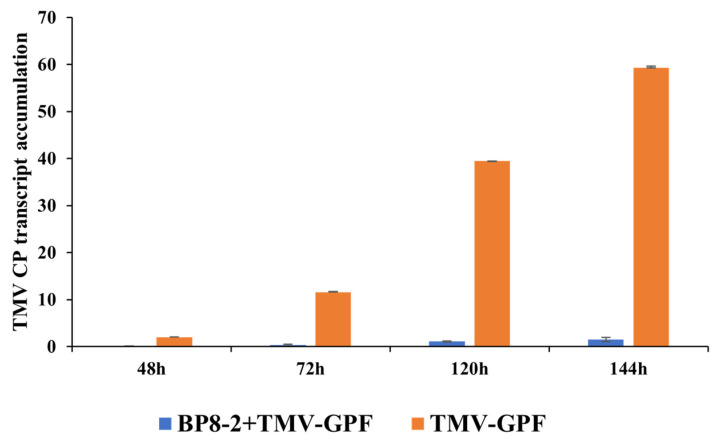
Accumulation of TMV-GFP in *N. benthamiana*.

For validating the inhibitory effect of BP8-2 on TMV expression and movement, 100 μg/mL BP8-2 was sprayed on *N. benthamiana*. After 24 h, 40 µL TMV-GFP (50 µL/mL) was inoculated on whole leaves. The results of real-time quantitative PCR (RT-qPCR) showed almost no TMV-GFP coat protein (CP) in the top leaves of tobacco treated with BP8-2 at 48 h, and the virus increased slowly with an increase in days (Figure 5). By contrast, those not treated with BP8-2 showed increased TMV-GFP CP content. These results indicate that BP8-2 has an inhibitory effect on TMV infection and movement.

### 2.7. Expression of Defence-Related Genes

**Figure 6:**
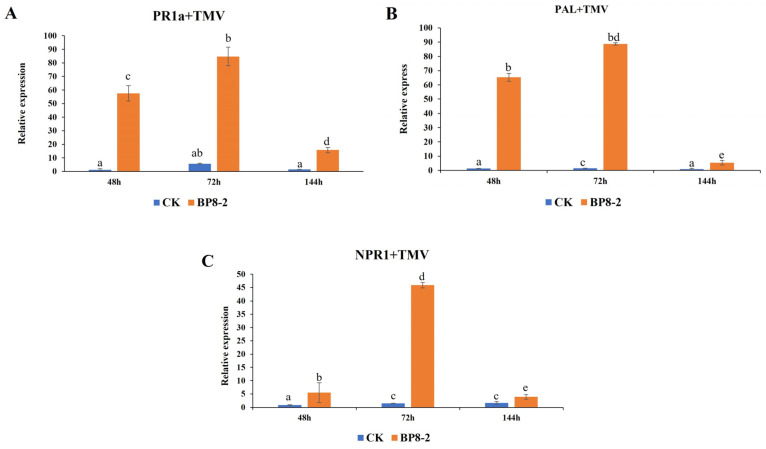
Expression analysis of defence-related genes in *N. benthamiana*. (**A**) *PR1a*; (**B**) *PAL*; (**C**) *NPR1*. Different lowercase letters indicate significant difference at *p* < 0.01 level by Duncan’s new multiple range test.

To detect whether BP8-2 causes the expression of defence-related genes, RT-qPCR was used to analyse the expression of *PR-1a*, *PAL*, and *NPR1* in BP8-2-treated tobacco leaves. As shown in Figure 6, after 24 h of spaying with BP8-2, followed by inoculation with TMV, the expression of the *PR1a*, *PAL*, and *NPR1* genes increased from 48 h, reaching a maximum at 72 h and then decreasing at 144 h. No significant increase in *PR1a*, *PAL*, or *NPR1* gene expression was observed in the TMV-treated tobacco leaves. At 48 h, the *PR1a*, *PAL*, and *NPR1* genes in the BP8-2 treatment group were significantly upregulated, and the upregulation amplitude was significantly higher than that in the control group, with the upregulation amplitudes being 48, 50, and 6 times, respectively. The findings demonstrated that BP8-2-induced high expression of different resistance response pathway genes.

## 3. Discussion

In this study, the flagellin BP8-2 gene was cloned using Ba168 genomic DNA as the template, and the fusion expression vector pCAMBIA1300-EGFP-BP8-2 was constructed. The fusion protein BP8-2 was expressed in *N. benthamiana* by employing the Agrobacterium-mediated tobacco transient expression system. The green fluorescence of BP8-2 was observed in the cell membrane and the nucleus of tobacco epidermal cells. The results of subcellular localisation were verified using RT-PCR technology, laying the foundation for further research on BP8-2. Effector proteins enter plant cells upon secretion by pathogenic bacteria and can fulfil their functions at different cellular locations. Ren et al. [32] discovered that the effector protein PITG_22926 secreted by *Phytophthora infestans* is located in the nucleus and exerts its function there. The relationship between the localisation of disease resistance genes and their functions has been extensively reported. Bai et al. [33] investigated barley MLA10 and found that the subcellular localisation of MLA10 in distinct subcellular regions also alters its role in causing barley cell death and inducing barley disease resistance. MLA10 localised in the cytoplasm can lead to programmed cell death, but the effect of inducing barley resistance to powdery mildew was not notable. Its localisation in the nucleus can induce barley resistance to powdery mildew but cannot cause programmed cell death. Du et al. [34] studied the function of the resistance protein R1 and found that the initiation of its resistance to potato late blight requires the co-localisation of R1 and AvR1 in the nucleus. Therefore, there is a crucial relationship between the resistance protein in different subcellular regions and the resistance response it induces. Whether BP8-2 localisation in different subcellular regions plays diverse functions in the plant defence response induced by BP8-2 in this study requires further testing.

As a result, there was stronger antiviral activity against TMV infection in *N. glutinosa* than in BP8-2 and ningnanmycin, with a protective effect of 73.98%. BP8-2 directly affected TMV movement and CP expression and induced high expression of the defence-related genes *PR-1a*, *PAL*, and *NPR1* in BP8-2-treated tobacco leaves. These results suggest that flagellin BP8-2 inhibits TMV by inducing resistance. Together with harpin, cryptogein, activator protein, and other avirulence proteins, flagellin BP8-2 acts as an elicitor of plant resistance and systemic responses [35,36]. Some studies have suggested that flagellin activates plant defence responses by participating in signal transduction pathways through receptor-like kinases and MAPK, and it is recognised by hosts as a PAMP factor of pathogen-related molecular patterns, inducing plants to produce a series of defence responses [37]. The active site is the N-terminal of *flg22*, composed of conserved 22-amino-acid polypeptides, which induce ROS outburst, nitric oxide production, callose deposition, and MAPK cascade reactions in plants [38]. These findings are consistent with those in this study. However, understanding the mechanism of BP8-2 inducing plant virus resistance requires further research.

The timely deployment of inducible defence responses is critical for effective resistance against pathogens. Flagellin, as a protein activator, has high thermal stability, good water solubility, broad-spectrum antibacterial activity, is non-immunogenic, and does not easily produce drug resistance. Flagellin can be used as an activating protein to stimulate the immune system of the plant so that the plant becomes resistant to various pathogens, and it is expected to become a new green biopesticide [12,39].

In conclusion, flagellin BP8-2 was localised in the cell membrane and nucleus, and the RT-PCR results showed that the BP8-2 gene could be stably expressed in tobacco leaf cells. Increased antiviral activity against TMV infection was created by inducing resistance. To the best of our knowledge, this is the first report on the application of flagellin for plant virus control. Antiviral mechanisms and crucial application points should be studied further. BP8-2 may play an important role in agriculture as it has low toxicity and is easily biodegradable and eco-friendly.

## 4. Material and Methods

### 4.1. Chemicals and Materials

Ningnanmycin AS (8%) was obtained from Deqiang Biology Co. Ltd. (Harbin, China). TaKaRa Ex Taq^®^ DNA Polymerase, restriction endonucleases KpnⅠ and XbaⅠ were obtained from Bao Biological Engineering Co., LTD (Dalian, China). TMV-GFP isolates were obtained from virus-infected plants of *N*. *benthamiana*. The seeds of *N*. *glutinosa* and *N. benthamiana* were cultivated in an insect-free greenhouse at 24 ± 1 °C. Experiments were conducted when the plants had grown 5–6 leaves. The plants, pCAMBIA1300-35S-EGFP, *B. amyloliquefaciens* Ba168, *Escherichia coli* DH5α, *Agrobacterium tumefaciens* C58C1, and prokaryotic expression of BP8-2 protein in *Escherichia coli* were maintained at the Laboratory of the College of Plant Protection, Gansu Agricultural University.

### 4.2. Virus Purification

Purification of TMV-inoculated *N. benthamiana* was performed as Gooding (1967) and Chen et al. (2019) have described [27,40].

### 4.3. Cloning of BP8-2 Gene

We designed gene-specific primers for BP8-2, BP8-2 F: 5′CTCTTATCCAAACATCTGAGGGTG 3′, and BP8-2 R: 5′ATTGAAGAACTTGCTTGCTGAGGCTGT 3′. PCR amplification was performed using *Bacillus subtilis* Ba168 DNA as the template, and the reaction mixture (25 μL) comprised DNA (1 μL), 10× PCR buffer (2.5 μL), dNTP Mix (2 μL), F primer (10 µmol/L; 1 μL), R primer (10 µmol/L; 1 μL), ddH_2_O (16.3 μL), and rTaq enzyme (0.2 μL). The cycling conditions were pre-denaturation at 94 °C for 4 min, denaturation at 94 °C for 30 s, annealing at 64 °C for 30 s, and extension at 72 °C for 45 s, for a total of 35 cycles, and extension at 72 °C for another 10 min. Next, 1% agarose gel electrophoresis was performed using 5 μL of the PCR product, and visualised by using an agel imaging system. The PCR product was purified using an anagarose gel purification kit and then recovered and ligated into the pMD19-T vector. After incubating at room temperature (22–23 °C) for 5 min, the ligation product was transformed into *Escherichia coli* DH5α-competent cells, transferred to LB (ampicillin-containing) liquid medium, and shaken overnight. White single colonies were randomly selected for overnight culture, positive colonies were selected for colony PCR testing, and plasmids were extracted. Positive plasmids based on restriction enzyme digestion results were sent to Qingke Biotechnology Co., Ltd. (Beijing, China) for sequencing analysis.

### 4.4. Construction and Sequencing of Recombinant Plasmid

The pCAMBIA1300-35S-EGFP vector and the flagellar protein BP8-2 gene fragment were digested simultaneously using two restriction endonucleases, KpnⅠ and XbaⅠ. The enzyme digestion system comprised 3 μL of the pCAMBIA1300-35S-EGFP vector or BP8-2, 5 mL 10× Cut-smart buffer, 1 μL KpnⅠ, 1 μL XbaⅠ, and 20 μL ddH_2_O, for a total volume of 40 μL. The digestion was conducted at 37 °C for 15 min. Subsequently, T4 ligase was used for ligation. The ligation system comprised 1 μL T4 ligase, 2 μL 10× T4 buffer, 1 μL of the digested pCAMBIA1300-35S-EGFP vector, 3 μL of the digested BP8-2 fragment, and ddH_2_O was added to make up a total volume of 10 μL. Ligation was performed at 25 °C for 10 min, followed by transformation into *Escherichia coli* DH5α-competent cells. Positive clones were selected for colony PCR and sequencing analysis. The correctly sequenced bacterial liquid containing the recombinant plasmid pCAMBIA1300-35S-BP8-2-EGFP was extracted using a plasmid mini-prep kit.

### 4.5. Subcellular Localisation and Transient Expression of BP8-2

The recombinant plasmid was then transformed into Agrobacterium C58C1. Both the Agrobacterium containing the pCAMBIA1300-35S-BP8-2-EGFP expression vector and those carrying the empty control vector pCAMBIA1300-35S-EGFP were cultured, and bacterial cells were collected (4000 rpm, 15 min) followed by removal of the supernatant. Next, 1 mL tobacco transformation solution (OD600 = 0.7~1.0) was added to resuspend Agrobacterium. After resuspension, the tobacco leaves were injected after standing at room temperature or 28 °C for 2 h.

Approximately 2–3 d after the injection of tobacco leaves with Agrobacterium, the lower epidermis of the tobacco leaves was peeled off, and the subcellular localisation of fused protein was observed using confocal microscopy. Images were captured simultaneously. Total RNA was extracted from the tobacco leaf for RT-PCR verification.

### 4.6. Purification of BP8-2 Protein by SDS-PAGE Electrophoresis

Flagellin BP8-2 was identified in *Bacillus amylolyticus*. Recombinant flagellin was obtained by introducing the *BP8-2* gene into *Escherichia coli*. After 16 h of induction at 16 °C with 0.5 mM IPTG, the 1000 mL culture expressing BP8-2 was harvested using centrifugation at 4000× *g* for 10 min at 4 °C. For cell lysate preparation, frozen cells were resuspended in 100 mL buffer (50 mM TRIS pH 8.0, 300 mM NaCl) containing 2% lysozyme (*v*/*v*) and completely homogenised using an ultrasonic cell disruptor (Ningbo Scientz Biotechnology, Ningbo, China). The cell lysate was then centrifuged at 12,000× *g* for 20 min at 4 °C to remove cell debris. BP8-2 was purified by using BeyoGold™ His-tag Purification Resin Protein purification kit (Beyotime Biotechnology, Shanghai, China). After washing, the bound fusion protein was eluted from the column with buffer A containing 300 mM imidazole. Then, 5× sample buffer (312.5 mM TRIS-HCl pH 6.8, 50% glycerol, 5% SDS, 0.05% bromophenol blue, 100 mM DTT) was prepared for mixing with protein fractions before running on 10% tricine SDS-PAGE gels. Protein bands were visualised using Coomassie brilliant blue R-250.

The purified protein concentration was determined using the Bradford method, the standard curve was made by using the protein standard solution (20 mg/mL BSA), and the protein concentration in the sample was obtained after the absorption value of the target protein (A595) was substituted.

### 4.7. Inhibitory Effect of BP8-2 on TMV Infection

The inhibitory effects of BP8-2 were examined using the half-leaf method. The virus (TMV at 6 × 10^−3^ mg/mL) was inhibited by mixing it with the BP8-2. As the positive control, 8% ningnanmycin was added using the same volume. After 10 min, 40 μL of the mixture was inoculated with a cotton swab on the left side of the tobacco leaves (*N. glutinosa*); the right side of the leaves was inoculated with the mixture of buffer solution and the virus as a control. Each of the three treatments (total of 18 leaves) was conducted. Each half of the leaf was smeared with 40 µL TMV extract. Local lesion numbers were recorded for 72–96 h after inoculation.

The inhibition rates of BP8-2 were calculated according to the formula

Inhibition rate (%) = [(C − T)/C] × 100%

where C is the average lesion number of local lesions of the negative control, and T is the average mean lesion number on the BP8-2-treated half-leaves.

### 4.8. Protective Effect of BP8-2 on TMV Infection

Next, 8% ningnanmycin was selected as the positive control, and 100 μg/mL BP8-2 and 8% ningnanmycin AS were gently smeared with cotton swabs onto the right side of the leaves of *Nicotiana glutinosa*, respectively. As a negative control, the buffered solution was spread on the left lobes of leaves of the same age. After 24 h, 40 µL TMV-GFP (50 µL/mL) was inoculated onto whole tobacco leaves, each containing three treatment groups repeated in triplicate. Each treatment used two leaves of *N. glutinosa* (a total of 18 leaves were recorded), and each inoculated leaf was washed with water after 10 min. The number of lesions on the tested leaves was investigated for 3–4 d.

### 4.9. Curative Effect of BP8-2 on TMV Infection

All growing leaves of *N. glutinosa* were mechanically inoculated with purified TMV (6 × 10^−3^ mg/mL). After 24 h, the BP8-2 was inoculated on the left leaf side, and the right side of the leaves of *N. glutinosa* was inoculated with a buffer solution as a control. As the positive control, 8% ningnanmycin was added with the same volume of BP8-2 on the left leaf side. The local lesion numbers were recorded for 72–96 h after viral inoculation. Three repetitions were conducted for each experiment.

### 4.10. Effects of BP8-2 on TMV Infection Movement

Here, 100 μg/mL BP8-2 was sprayed onto *N. benthamiana.* After 24 h, 40 µL of TMV-GFP (50 µL/mL) was inoculated onto the whole leaves. The leaves were then washed with water and dried. The expression of TMV-GFP was observed using RT-qPCR at 48 h, 72 h, 120 h, and 144 h, respectively.

To detect the content of TMV CP at different time points, total RNA extracted from *N. benthamiana* was treated with BP8-2, and the control was prepared using RNAiso Plus (Takara Biotechnology, Dalian, China) and subjected to reverse transcription to cDNA (Tiangen, Beijing, China). Real-time quantitative PCR (RT-qPCR) was performed using SYBR^®^ Green Pro Taq HS (Agbio, Changsha, China), following the supplier’s guidelines. The conditions were as follows: 95 °C for 30 s, 59 °C for 30 s, and 60 °C for 40 s. Actin was used as the reference gene, with three independent biological replicates. TMV CP and actin gene expression levels were analysed using an ABI StepOne Plus (Applied Biosystems, Foster City, CA, USA). The 2^−ΔΔCt^ method was used for calculation. Primers used for qRT-PCR are listed in Table 2.

### 4.11. Detection of the Expression of Defence-Related Genes by Real-Time Quantitative PCR

After treating the tobacco as described in Section 4.7, the expression levels of *PR-1a*, *PAL*, and *NPR1* in the BP8-2-treated tobacco leaf were measured using RT-qPCR at 48, 72, and 144 h, respectively. The primer sequences used for RT-qPCR are listed in Table 3.

### 4.12. Statistical Analysis

All data are presented as the mean ± the standard error of the mean (SEM). All experiments were performed in at least three biological replicates. Statistical analyses were conducted using SPSS 21.0. Differences were considered statistically significant at *p* < 0.05.

## Figures and Tables

**Table 1:** Antiviral activity of protein BP8-2 against TMV.

Drug	Inhibition Effect (%)	Protection Effect (%)	Curative Effect (%)
100 μg/mL BP8-2	75.91 ± 5.33 a	77.45 ± 5.17 a	68.15 ± 5.93 a
1000 times 8%Ningnanmycin SL	63.92 ± 6.13 aA	65.20 ± 2.02 aA	61.67 ± 3.98 aA

Values are presented as mean ± SE. Different uppercase and lowercase letters in the same column indicate significant differences at the *p* < 0.01 or *p* < 0.05 level by Duncan’s new multiple range test.

**Table 2:** Primer sequences used for the RT-qPCR analysis of TMV CP expression.

Gene Name	Forward Primer Sequences (5′-3′)	Reverse Primer Sequences (5′-3′)
*Actin*	CTTGAAACAGCAAAGACCAGC	CTTGAAACAGCAAAGACCAGC
*TMV CP*	GACCTGACAAAAATGGAGAAGATCT	GAAAGCGGACAGAAACCCGCTG

**Table 3:** Primer sequences used for RT-qPCR.

Gene Name	Forward Primer Sequences (5′-3′)	Reverse Primer Sequences (5′-3′)
*PR1a*	GATGCCCATAACACAGCTCG	GATGCCCATAACACAGCTCG
*PAL*	ATTGCTGGTTTGCTCACTGG	TCCTTAGGCTGCAACTCGAA
*NPR1*	GATACACGGTGCTGCATGTT	AAGCCTAGTGAGCCTCTTGG

## Data Availability

Data are contained within the article.
